# Laparoscopic Resection of a Gastric Plasma Cell Granuloma: A Case Report

**DOI:** 10.1155/2012/589682

**Published:** 2012-04-17

**Authors:** Christopher Horn, Huw G. Jones, Gareth Leopold, Anna Mainwaring, Ashraf Rasheed

**Affiliations:** ^1^Department of Oesophagogastric Surgery, Royal Gwent Hospital, Newport, South Wales NP20 2UB, UK; ^2^Department of HPB Surgery, University Hospital of Wales, Heath Park, Cardiff CF14 4XW, UK

## Abstract

Plasma cell granuloma, also known as inflammatory myofibroblastic tumour or inflammatory pseudotumour, is a nonneoplastic process characterized by an unregulated growth of inflammatory cells. It most commonly occurs in the lung and upper respiratory tract, and only six other cases of gastric plasma cell granuloma exist. There are no other cases of intragastric laparoscopic resection of this type of lesion. Here, we present a case of a 60-year-old gentleman who had gradual onset epigastric discomfort and was thought to have a gastrointestinal stromal tumour on gastroscopy. Subsequent imaging and laparoscopic transgastric resection of the lesion confirmed the presence of a plasma cell granuloma. We discuss the aetiologies, presentation, investigation, and treatment of this rare disorder and make recommendations on the management.

## 1. Case Report

A 60-year-old gentleman presented with gradually worsening epigastric discomfort and weight loss over a six-month period. Due to continuing symptoms following antireflux therapy, an urgent oesophagogastroduodenoscopy was arranged. An ulcerated lesion was demonstrated in the proximal lesser curvature of the stomach, which was thought to be a Gastrointestinal Stromal Tumour due to its appearance (GIST).

**Figure 1:**
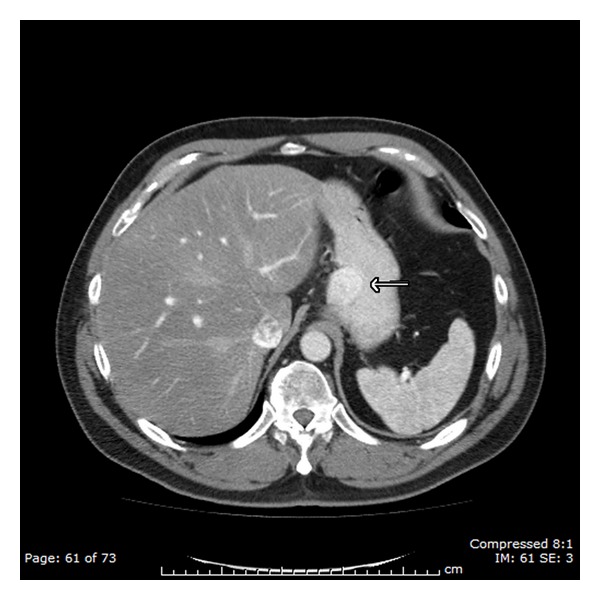
Axial CT scan of the abdomen, demonstrating a 36 × 34 mm submucosal circumscribed lesion on the lesser curvature of the stomach (arrow).

An urgent double-contrast CT scan of the abdomen was arranged (Figure 1). This demonstrated a 36 mm by 34 mm submucosal circumscribed lesion high on the lesser curvature of the stomach, in close proximity to the gastro-oesophageal junction. There was also minor perigastric lymphadenopathy, but no metastatic lesions were visible in the liver, lungs, or bones. A staging laparoscopy confirmed minor extension into the serosa, but no obvious extragastric component.

The patient was discussed in the local oesophagogastric multidisciplinary team meeting, and along with the patients' wishes, primary surgery was suggested as the best option, due to the likelihood of a diagnosis of GIST.

**Figure 2:**
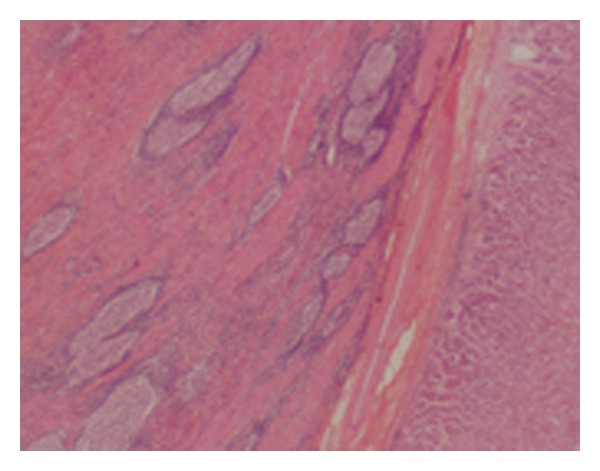
Histological section of the plasma cell granuloma (haematoxylin and eosin, ×20).

**Figure 3:**
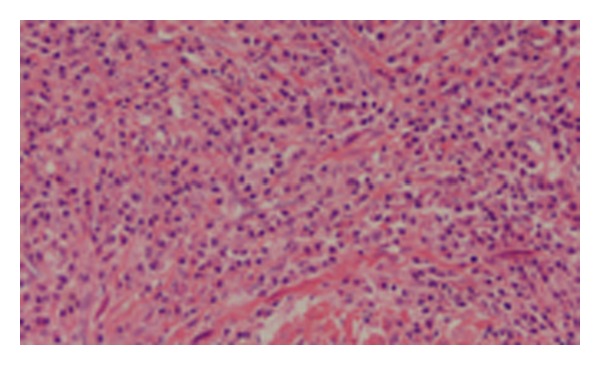
Histological section of the plasma cell granuloma (haematoxylin and eosin, ×400).

An intragastric laparoscopic resection was performed within four weeks of the initial presentation. This was chosen over a proximal gastrectomy in order to preserve the antireflux system of the patient. A 70 × 40 × 20 mm specimen was excised and sent for histopathology (Figures 2 and 3). The patient had an uneventful recovery and was discharged from hospital after 5 days.

The histopatholgy report demonstrated a good resection margin of over 1 mm. The specimen consisted of mature chronic inflammatory cells, mainly plasma cells and reactive-type lymphoid follicles. Alk-1 immunostaining identified occasional scattered larger mononuclear cells with open nuclei and occasional nucleoli. This was consistent with a plasma cell granuloma, a nonneoplastic polyclonal proliferation of mature plasma cells.

The patient is now symptom-free and has had regular follow-up CT scans of his abdomen, and a gastroscopy, which has not demonstrated any recurrence within 18 months of the procedure.

## 2. Discussion

Plasma cell granuloma is referred to as several other names in the literature, including inflammatory myofibroblastic tumour, inflammatory pseudotumour, fibrohistiocytoma, and fibroxanthoma. It is a rare disease that is characterised by a nonneoplastic proliferation of mature plasma cells in a fibrovascular background [1]. Tada et al. [2] first described the condition in 1973. They most commonly occur in the lungs and upper respiratory tract, and extrapulmonary lesions are extremely rare [3]. There are documented cases in the spleen, stomach, pancreas, liver, intracranial and intra-spinal space, thyroid, larynx, orbit, heart, and kidney [4].

There have been very few cases of gastric plasma cell granuloma, and there are no other cases that have been primarily treated with laparoscopic surgery. It is generally considered a benign condition, with an inflammatory component, but has been reported in association with gastric cancer [2]. Kim et al. [5] reported a case of a plasma cell granuloma with peritoneal dissemination in a young adult. They stated that the lesion extended beyond the gastric mucosa into surrounding organs, including oesophagus, duodenum, spleen, and peritoneal cavity.

There is very little data on the aetiology, pathogenesis and the most effective treatment for this disorder; therefore, the prognosis of the condition is difficult to predict. The most common treatment for these lesions is complete excision although this is not always possible. Radiotherapy [6] and steroid therapy [7, 8] have been suggested, with a few isolated successes; however, further data is needed before these therapies can be safely advocated as an alternative to excision.

Here, we present a case of a 60-year-old gentleman with a six-month history of worsening epigastric pain. Due to the possibility of the presence of a malignant lesion, such as a GIST, it was urgently and thoroughly investigated using a combination of noninvasive imaging (double-contrast CT scan of the abdomen) and invasive investigations (gastroscopy and diagnostic laparoscopy). Due to the continuing diagnostic doubt regarding the exact nature of the lesion, an intragastric laparoscopic procedure was undertaken in order to remove the lesion with minimal disruption to organ function. The surprising diagnosis was of a rare gastric plasma cell granuloma. The patient recovered well, with a resolution of symptoms, and no recurrence was found at 18-month follow-up.

## 3. Conclusions

Nonneoplastic tumours of the stomach are rare and can present with very similar symptoms to malignant lesions, such as epigastric discomfort and microcytic anaemia. The diagnosis should be kept in mind, especially for solitary lesions with no evidence of metastases. Due to the rarity of gastric plasma cell granuloma, each new case should be recorded to allow a better understanding of the condition. We advocate the use of intragastric laparoscopic resection of these lesions in order to provide a diagnosis and avoid complications associated with more extensive surgery, such as bleeding, loss of organ function, and acid reflux disease.
